# The brain’s conversation with itself: neural substrates of dialogic inner speech

**DOI:** 10.1093/scan/nsv094

**Published:** 2015-07-20

**Authors:** Ben Alderson-Day, Susanne Weis, Simon McCarthy-Jones, Peter Moseley, David Smailes, Charles Fernyhough

**Affiliations:** ^1^Department of Psychology, Durham University, Durham, UK,; ^2^Department of Cognitive Science, Macquarie University, Australia,; ^3^Department of Psychiatry, Trinity College Dublin, Ireland, and; ^4^School of Psychology, University of Central Lancashire, Preston, UK.

**Keywords:** monologue, dialogue, fMRI, auditory verbal hallucinations, covert speech

## Abstract

Inner speech has been implicated in important aspects of normal and atypical cognition, including the development of auditory hallucinations. Studies to date have focused on covert speech elicited by simple word or sentence repetition, while ignoring richer and arguably more psychologically significant varieties of inner speech. This study compared neural activation for inner speech involving conversations (‘dialogic inner speech’) with single-speaker scenarios (‘monologic inner speech’). Inner speech-related activation differences were then compared with activations relating to Theory-of-Mind (ToM) reasoning and visual perspective-taking in a conjunction design. Generation of dialogic (compared with monologic) scenarios was associated with a widespread bilateral network including left and right superior temporal gyri, precuneus, posterior cingulate and left inferior and medial frontal gyri. Activation associated with dialogic scenarios and ToM reasoning overlapped in areas of right posterior temporal cortex previously linked to mental state representation. Implications for understanding verbal cognition in typical and atypical populations are discussed.

## Introduction

Inner speech—the experience of silent, verbal thinking—has been implicated in many cognitive functions, including problem-solving, creativity and self-regulation (Morin, 2009; Fernyhough, 2013; Alderson-Day and Fernyhough, 2015a), and disruptions to the ‘internal monologue’ have been linked to varieties of pathology, including hallucinations and depression (Frith, 1992; Nolen-Hoeksema, 2004). Enhanced understanding of inner speech hence has implications for understanding of both typical and atypical cognition. Although interest in inner speech has grown in recent years (Morin *et al*., 2011; Williams *et al*., 2012; Fernyhough, 2013), conceptual and methodological challenges have limited what is known about the neural processes underpinning this common experience.

Most neuroimaging studies to date have operationalized inner speech as a unitary phenomenon equivalent to a first-person monologue (Hinke *et al*., 1993; Simons *et al*., 2010). Methods of eliciting inner speech have typically involved either subvocal recitation (e.g. covertly repeating ‘You are a *x*’ in response to a cue; McGuire *et al*., 1995) or prompting participants to make phonological judgements about words using inner speech (such as which syllable to stress in pronunciation; Aleman *et al*., 2005). Such studies have shown recruitment during inner speech of areas associated with overt speech production and comprehension, such as left inferior frontal gyrus (IFG), supplementary motor area (SMA) and the superior and middle temporal gyri (McGuire *et al*., 1996; Shergill *et al*., 2002; Aleman *et al*., 2005).

However, inner speech is a complex and varied phenomenon. In behavioural studies, everyday inner speech is often reported to be involved in self-awareness, past and future thinking and emotional reflection (D’Argembeau *et al*., 2011; Morin *et al*., 2011), while in cognitive research, inner speech appears to fulfill a variety of mnemonic and regulatory functions (e.g. Emerson and Miyake, 2003; see Alderson-Day and Fernyhough, 2015a, for a review). Vygotsky (1987) posited that inner speech reflects the endpoint of a developmental process in which social dialogues, mediated by language, are internalized as verbal thought. Following from this view, the subjective experience of inner speech will mirror the external experience of communication and often have a dialogic structure (Fernyhough, 1996, 2004), involving the co-articulation of differing perspectives on reality and, in some cases, representation of others’ voices. Evidence for the validity of these distinctions is provided by findings from a self-report instrument, the varieties of inner speech questionnaire (VISQ: McCarthy-Jones and Fernyhough, 2011). Studies with student samples have documented high rates of endorsement (>75%) for inner speech involving dialogue rather than monologue, alongside a number of other phenomenological variations (Alderson-Day *et al*., 2014; Alderson-Day and Fernyhough, 2015b).

Recognizing this complexity of inner speech, particularly its conversational and social features, is important both for ecological validity (Fernyhough, 2013) and for understanding atypical cognition (Fernyhough, 2004). Auditory verbal hallucinations (AVH) have been proposed to reflect misattributed instances of inner speech (Bentall, 1990; Frith, 1992), but studies inspired by this view have arguably relied on a relatively impoverished, ‘monologic’ view of inner speech. In the context of a growing recognition of social and conversational dimensions of AVH (Bell, 2013; Ford *et al*., 2014), knowing more about the heterogeneity of inner speech could enhance AVH models (Jones and Fernyhough, 2007).

Almost no data exist on the neural basis of dialogic or conversational inner speech, and what there is has largely focused on imagining words or sentences spoken in other voices (often referred to as ‘auditory verbal imagery’). For example, Shergill *et al*. (2001) asked participants either to silently rehearse sentences of the form ‘I like *x*…’ in their own voice (inner speech) or to imagine sentences spoken in another voice in the second or third person (auditory verbal imagery). While sentence repetition was associated with activation of left IFG, superior temporal gyrus (STG), insula and the SMA, imagined speech in another person’s voice recruited a bilateral frontotemporal network, including right IFG, left pre-central gyrus and right STG. Similarly, in an AVH study by Linden *et al*. (2011), auditory imagery for familiar voices, such as conversations with family members, was associated with bilateral activation in IFG, superior temporal sulcus (STS), SMA and anterior cingulate cortex in healthy participants.

Research on overt conversational processing has also implicated a bilateral network including right frontal and temporal homologues of left-sided language regions. For example, Caplan and Dapretto (2001) compared judgements for logical and contextual violations of conversations in an functional magnetic resonance imaging (fMRI) task. Whereas logic judgements were associated with a left-sided Broca–Wernicke network, judgements about pragmatic context recruited right inferior frontal and middle temporal gyri, along with right prefrontal cortex (PFC). The involvement of right frontotemporal regions in pragmatic language processing is supported by evidence of selective impairments in prosody, humour and figurative language in cases of right-hemisphere damage (Mildner, 2007).

Finally, two recent studies by Yao *et al*. (2011; 2012) have indicated a specific role for right auditory cortex in the internal representation of other voices. In a study of silent reading, Yao *et al*. (2011) examined activation of left and right auditory cortex when participants read examples of direct and indirect speech (e.g. ‘The man said ‘I like cricket’’ *vs* ‘The man said that he likes cricket’). Reading of direct speech was specifically associated with activation in middle and posterior right STS compared with indirect speech. The same areas were also active in a second study (Yao *et al*., 2012) when participants listened to examples of direct speech read in a monotonous voice, but that was not the case during listening to indirect speech. Yao *et al*. argued that the activation of these regions during silent reading and listening to monotonous direct speech might reflect an internal simulation of the suprasegmental features of speech, such as tone and prosody.

Taken together, these findings suggest that dialogic forms of inner speech are likely to draw on a range of regions beyond a typical left-sided perisylvian language network, including the right IFG, right middle temporal gyrus (MTG) and the right STG/STS. Following Shergill *et al*. (2001) and, to a lesser degree, Yao *et al*. (2011), it could be hypothesized that the involvement of these regions is required for the simulation of other people’s voices to complement one’s own inner speech. On such a view, dialogic inner speech could be conceptualized simply as monologic inner speech plus the phonological representation of other voices, leading to recruitment of voice-selective regions of right temporal cortex.

However, generating an internal conversation requires more than simply mimicking the auditory qualities of the voices involved. First, dialogic inner speech could draw on theory-of-mind (ToM) capacities, requiring not only just the representation of a voice but also the sense and intention of a plausible and realistic interlocutor. If dialogic inner speech utilized such processes, then it should be possible to identify recruitment of typical ToM regions, including medial PFC (mPFC), posterior cingulate/precuneus and the temporoparietal junction (TPJ) area, encompassing posterior STG, angular gyrus and inferior parietal lobule (Spreng *et al*., 2009). Right TPJ has been associated with ToM in a number of fMRI and positron emission tomography (PET) studies, mostly based on false-belief tasks (Saxe and Powell, 2006), while left TPJ has been linked to mental state representation (Saxe and Kanwisher, 2003) and understanding of communicative intentions (Ciaramidaro *et al*., 2007). A view of dialogic inner speech as drawing on ToM capacities would suggest that it should be associated with established ToM networks and posterior temporoparietal cortex, in addition to frontotemporal regions associated with voice representation.

A second key difference between dialogue and monologue concerns their structure and complexity. Generating an internal dialogue involves representational demands that are absent from sentence repetition or subvocal rehearsal. Whereas, in monologue, a single speaker’s voice or perspective is sufficient, in dialogue more than one perspective must be generated, maintained and adopted on an alternating basis (Fernyhough, 2009). Internally simulating a conversation could also involve imagination of setting, spatial position and other details that distinguish interlocutors. Therefore, any differences observed between dialogic and monologic inner speech may not reflect representation of other voices or agents, so much as indexing the requirement to generate and flexibly switch between conversational positions and situations ‘in the mind’s eye’. If dialogic inner speech depended on such skills, it might be expected to recruit areas more typically associated with the generation and control of mental imagery, such as middle frontal gyrus (MFG), precuneus and superior parietal cortex (Zacks, 2007; McNorgan, 2012).

There are therefore reasons to believe that the production of dialogic inner speech will differ from monologic examples of the same process in three ways: recruitment of regions involved in representing other voices, involvement of ToM resources to represent other agents and the activation of brain networks involved in the generation and control of mental imagery. To test these predictions, we employed a new fMRI paradigm for eliciting monologic (i.e. verbal thinking from a single perspective) and dialogic inner speech, so that the neural correlates of the two can be compared.

To investigate the cognitive processes involved in dialogic inner speech, we used a conjunction analysis (Price and Friston, 1997) to compare dialogue-specific activation with two other tasks: a ToM task (Walter *et al*., 2004) and a novel perspective-switching task. The ToM task was chosen because it included non-verbal scenarios requiring inferences about communication and the representation of other agents’ intentions; in this way, any conjunction between dialogue and ToM should not reflect overlaps in the processing of verbalized language. The perspective-switching task was developed to match the switching and imagery-generation demands of the dialogic task, while avoiding the inclusion of social agents, which feature in many existing perspective-switching tasks. Conjunctions observed between the perspective-switching and dialogic tasks should therefore reflect similarities in structure and task demands, rather than representations of agents and mental states tapped in the ToM task. We predicted that (i) dialogic inner speech—in contrast to a monologic control condition—would activate not only right-hemisphere language homologue regions such as right IFG, MTG and STG but also areas typically associated with ToM processing, such as the TPJ and (ii) any further differences between dialogic and monologic scenarios would overlap with networks associated with perspective switching and mental imagery, such as the MFG or the superior parietal lobule.

## Materials and methods

### Participants

Twenty-one individuals [6 male; age *m*(s.d.)* = *24.38 (6.73) years] were recruited from university settings. All participants were right-handed, native English speakers with normal or corrected-to-normal vision. No participants reported any history of cardiovascular disease, neurological conditions or head injury. Participants received either course credit or a gift voucher. All procedures were approved by the local university ethics committee.

### Scanning materials and procedure

Participants completed three tasks in the scanner: inner speech, ToM and perspective-switching (followed by an anatomical scan). Each task was preceded by a single practice trial. All stimuli were presented using E-Prime 2.0 (Schneider *et al*., 2002). Participants viewed stimuli by looking upwards at a mirror directed at a monitor (Cambridge Research Systems Ltd. BOLDscreen MR Safe display; 1920 × 1200 resolution, refresh rate 60 Hz) placed behind the scanner bore. Button press responses (all right-handed) were collected using a fiber-optic response button box (Psychology Software Tools).

#### Inner speech

Participants were presented with a written description of a scenario involving either dialogue or monologue and were asked to generate inner speech in that scenario until they saw a cue to stop. Dialogic scenarios involved conversations and interviews with familiar people (Table 1). Monologic scenarios were matched to dialogic scenarios for their content and setting, but only included a single speaker. Instructions were presented for 10 s, followed by a fixation cross (the cue for inner speech) for 45 s and an intertrial interval of 3–5 s (including a stop signal for 2 s). In total, five dialogic and five monologic scenarios were presented. At the end of the scanning session, participants were asked to rate out of 100 (i) how vividly they imagined the scenarios, (ii) the vividness of any visual imagery they used during the task and (iii) the everyday characteristics of their own inner speech, using the VISQ (McCarthy-Jones and Fernyhough, 2011). The imagery self-ratings were included to check task compliance and to provide a control indicator of how much participants drew on visual (rather than verbal) imagery during the task. The VISQ was included for exploratory analysis of how individual differences in everyday inner speech may have affected task performance and related brain activations. It includes four subscales: dialogic inner speech (items include, e.g. ‘I talk back and forward to myself in my mind about things’), evaluative/motivational inner speech (e.g. ‘I think in inner speech about what I have done, and whether it was right or not’), other people in inner speech (e.g. ‘I experience the voices of other people asking me questions in my head’) and condensed inner speech (e.g. ‘I think to myself in words using brief phrases and single words rather than full sentences’). The VISQ has been shown to have good internal and test–retest reliability (McCarthy-Jones and Fernyhough, 2011; Alderson-Day *et al*., 2014).

**Table 1.** Dialogic and monologic scenarios in the inner-speech task

Scenario	Dialogic	Monologic
A visit to your old school	Conversation with a teacher	Making a speech to students
A job interview	Talking to the interviewer	Doing a presentation
Calling a relative	Conversation with relative	Leaving a voicemail
Being in a documentary	Doing an interview	Speaking to camera
Meeting the Prime Minister	Interviewing the PM	Suggesting a new law

#### Theory-of-mind

Using a cartoon-based ToM task from Walter *et al*. (2004), participants viewed a sequence of three cartoons depicting a simple story (‘Story’ phase) and were then prompted to choose the logical end of the story from three options (‘Choice’ phase). Stories either required deciphering of actors’ intentions (e.g. pointing to see if a seat was free) or reasoning about physical causality (e.g. a football breaking some bottles). To examine ToM skills relevant to inner speech, the ‘communicative intention’ condition from Walter *et al*. (2004) was used, as compared with the physical reasoning control condition. ‘Story’ phase images were presented sequentially for 3 s each, followed by the ‘Choice’ phase for 7 s and a jittered intertrial interval of 7–11 s. A total of 10 ToM stories and 10 physical reasoning stories were presented in a random order. Participants indicated which image completed the story (A, B or C) using a button box, and their percentage accuracy was recorded.

#### Visual perspective switching

The timing and structure of the perspective-switching task was designed to match the inner-speech task. Participants first viewed an instruction page (10 s) describing a visual scene or object and asking them to imagine it from a particular perspective, e.g. ‘Imagine a train viewed from the outside. Try to picture what it looks like in your mind.’ Underneath, this was followed by an instruction to either switch perspective when prompted by a cue (the ‘Switch’ condition) or to maintain the image from single perspective until prompted to stop (the ‘Stick’ condition). In the Switch condition, the instruction page was followed by a 45 s imagery phase, in which every 7 s a cue appeared (either ‘OUTSIDE’ or ‘INSIDE’, 2 s presentation). In the Stick condition, cues appeared with the same regularity but only from one perspective (i.e. only ‘INSIDE’). After scanning, participants rated how vividly they had imagined each scene/object, and how easy they found switching between different viewpoints (rated out of 100).

### Mock scanner behavioural task

Production of inner speech is difficult to verify objectively, leaving open the possibility that any differences observed between dialogic and monologic scenarios might not reflect underlying inner speech processes. To explore this further, we ran a *post* *hoc* behavioural study in a mock MRI scanner that replicated the layout, conditions and stimulus setup of the 3T scanner used for imaging. A separate set of 20 participants [2 male; age *m*(s.d.)* = *19.65 (1.31) years] attempted the original inner-speech task and then rated a variety of phenomenological characteristics for each dialogic and monologic scenario (see Supplementary Materials for an example response sheet). Specifically, participants rated each scenario for its (i) overall vividness, (ii) presence of inner speech, (iii) presence of visual imagery, (iv) vividness of one’s own voice, (v) vividness of other voices, and (vi) the number of times there was a ‘switch’ in perspective, voice or role (items 1–5 were rated as percentages).

Following this, participants also attempted a novel version of the inner-speech task that included articulatory suppression, a commonly used secondary task that is thought to interfere with inner speech use (e.g. Baddeley *et al.*, 1984; Williams *et al*., 2012). Specifically participants were asked to attempt the inner-speech task again but while repeating a different day of the week, out loud, for the duration of each scenario. The idea of this was to test whether engaging with the inner-speech task really did require use of inner speech to be performed successfully. To minimize effects of repeating the same scenarios, participants were encouraged to modify each situation (i.e. imagine speaking to a different relative) and only had to imagine scenarios for half the original time (22.5 s).

### fMRI acquisition

All data were acquired at Durham University Neuroimaging Centre using a 3T Magnetom Trio MRI system (Siemens Medical Systems, Erlangen, Germany) with standard gradients and a 32-channel head coil. T2*-weighted axial echo planar imaging (EPI) scans were acquired parallel to the anterior/posterior commissure line with the following parameters: field of view (FOV) = 212 × 212 mm, flip angle (FA) = 90°, repetition time (TR) = 2160 ms, echo time (TE) = 30 ms, number of slices (NS) = 35, slice thickness (ST) = 3.0 mm, interslice gap = 0.3 mm, matrix size (MS) = 64 × 64. Images for each task were collected as separate runs (280 volumes each per run). For each participant, an anatomical scan was acquired using a high-resolution T1-weighted 3D-sequence (NS: 192; ST: 1 mm; MS: 512 × 512; FOV: 256 × 256 mm; TE: 2.52 ms; TR: 2250 ms; FA 9°).

### Data analysis

All analyses were conducted using Statistical Parametric Mapping (SPM), version 8 (Wellcome Department of Cognitive Neurology, London, UK) implemented in MATLAB (2012b) (The Mathworks Inc).

Images were realigned to the first image to correct for head movement. After realignment, the signal measured in each slice was shifted in time relative to the acquisition time of the middle slice using a sinc interpolation to correct for different acquisition times. Volumes were then normalized into standard stereotaxic anatomical MNI-space using the transformation matrix calculated from the first EPI-scan of each subject and the EPI-template. The default settings for normalization in SPM8 with 16 non-linear iterations and the standard EPI-template supplied with SPM8 were used. The normalized data with a resliced voxel size of 3 × 3 × 3mm were smoothed with a 6 mm full width half maximum (FHWM) isotropic Gaussian kernel to accommodate intersubject variation in brain anatomy. The time-series data were high-pass filtered with a high-pass cutoff of 1/128 Hz and first-order autocorrelations of the data were estimated and corrected for. The first four volumes of each run were discarded to allow for equilibrium of the T2 response. Movement parameters from the realignment phase were visually inspected for outliers and included as regressors for single-subject (first level) analyses.

Single-subject analyses were conducted using a general linear model. The inner-speech and perspective-switching tasks were modelled as a block design with an instruction phase (4 volumes) and imagery phase (17 volumes). For the inner-speech task, three conditions were modelled in the analyses: monologic inner speech (17v), dialogic inner speech (17v) and the instruction phase (4v). The perspective-switching task was modelled in an identical way, but with Switch and Stick conditions instead of dialogic and monologic. The expected hemodynamic response at stimulus onset was modelled as a block design, convolved with a canonical hemodynamic response function. Following Walter *et al*. (2004), the ToM task was modelled as an event-related design with four regressors: ToM-Story, ToM-Choice, Physical-Story and Physical-Choice. Subsequently, parameter estimates of the regressor for each of the different conditions were calculated from the least mean squares fit of the model to the time-series. ‘Story’ and ‘Choice’ regressors on the ToM task were combined within each condition for the generation of contrast images (Walter *et al*., 2004).

For the inner-speech task, differences between parameter estimates for dialogic and monologic inner speech were tested within-subjects at the individual level, then tested at the group level with a one sample *t*-test. Comparisons of dialogic and monologic conditions with baseline were also made to provide further information on each condition’s neural correlates. The same procedure was applied for key comparisons on the ToM task and perspective-switching task (ToM Reasoning > Physical Reasoning and Switch > Stick, respectively). The contrasts between dialogic and monologic inner speech, ToM Reasoning and Physical Reasoning and Switch and Stick conditions were then used in a conjunction analysis to assess shared components of each task.

Because differences between dialogic and monologic inner speech were expected to be relatively small, we chose a cluster correction with a higher sensitivity to small sample sizes in comparison to the SPM cluster correction. A cluster extent threshold method (Slotnick *et al*., 2003; Slotnick and Schacter, 2004) was used to identify groups of contiguous voxels that were active at a value of *P* < 0.05, corrected for multiple comparisons. A Monte Carlo simulation with 10 000 iterations was used to estimate cluster thresholds based on the voxel-wise probability of a Type 1 error. For a voxel-wise error of *P* < 0. 01, a cluster of 11 or more voxels was required for *P* < 0.05, corrected for multiple comparisons. For a voxel-wise error of *P* < 0.001, clusters of 6 or more voxels were required for *P* < 0.05, corrected. As the latter criterion has been recommended to avoid false positives (Woo *et al*., 2014), the results reported later are all significant at *P* < 0.05 (corrected) based on a voxel-wise error of *P* < 0.001, unless otherwise stated. MNI voxel positions were converted into equivalent Talairach and Tournoux (1988) co-ordinates in MATLAB for anatomical labelling. All structure and Brodmann areas (BA) were labelled using the Talairach Daemon applet (Lancaster *et al*., 2000). Brain images were generated using SPM and MRICron (Rorden *et al*., 2007).

## Results

Two participants were excluded from the analyses due to movement during the inner-speech task. Thus, the results later display data from a sample of 19 participants (5 male, age *m*(s.d.)* = *24.63 (7.01) years).

### Inner speech

Table 2 displays the contrast between dialogic and monologic inner speech (all clusters at *P* < 0.05, corr.). Significantly increased activation for dialogic compared with monologic inner speech was evident in STG bilaterally, left inferior and medial frontal gyri and a collection of posterior midline structures, including the left precuneus and right posterior cingulate. The opposite contrast, Monologic > Dialogic inner speech, did not identify any significant activations. Compared with baseline, dialogic inner speech was associated with significantly increased activation in left posterior insula (*x* = −39; *y* = −18, *z* = 7; *t* = 4.38, *P* < 0.05, corr.) only. At more liberal threshold levels (when the cluster extent was thresholded based on a voxel-wise error of *P* < 0.01), both dialogic and monologic inner speech were associated with left-hemisphere activation compared with baseline, including the left IFG, medial frontal gyrus, insula and caudate.

**Table 2.** Regions activated significantly more during dialogic inner speech as compared with monologic inner speech (all *P* < 0.05, corrected, minimal cluster size 6 voxels.)

	BA	*x*	*y*	*z*	*t*	No. of voxels
L precuneus	31	−15	−58	34	7.44	566
R superior temporal gyrus	41	50	−26	16	6.76	128
R superior temporal gyrus	13	42	−47	21	6.70	16
R superior temporal gyrus	13	48	−41	22	6.49	22
R cingulate gyrus	23	6	−17	32	6.32	128
L medial frontal gyrus	9	0	35	34	6.28	158
L inferior frontal gyrus	47	−24	29	−11	5.87	14
R posterior cingulate	30	21	−47	12	5.49	10
R posterior cingulate	31	24	−58	15	5.49	27
L STG/insula	13	−42	−21	5	5.43	17
L cerebellum		−30	−48	−17	5.08	28
L middle occipital gyrus	18	−27	−82	8	4.92	8
L thalamus		−21	−27	8	4.88	13
L superior temporal gyrus	13	−45	−46	−17	4.59	6
R pre-central gyrus	9	36	6	31	4.23	11
R middle temporal gyrus	37	48	−60	−1	4.03	6

Self-ratings for vividness of inner speech scenarios were high (*m* = 73.42, s.d. = 13.13). Vivid visual imagery was also reported, although this tended to vary considerably across participants (*m* = 58.68, s.d. = 27.73, range = 0–100).

### Theory-of-mind

The contrast between ToM and physical reasoning was associated with significant activation in anterior and posterior STG bilaterally, along with midline activation centring on left precuneus (Table 3). Although left STG activity separated into anterior and posterior clusters, right STG activation was centred on posterior areas close to the TPJ but evident all along the gyrus. In contrast, physical reasoning compared with ToM reasoning showed significantly greater recruitment of the left anterior lobe of the cerebellum (−27, −48, −12, *t* = 5.97), right cuneus (15, −82, 5; *t* = 5.73), right caudate (27, −41, 18; *t* = 3.88), left post-central gyrus (−45, −28, 36; *t* = 4.60) and left lingual gyrus (−21, −80, −8; *t* = 4.10). Performance on the ToM task was acceptable (Accuracy *m* = 84.21%, s.d. = 10.03%, range = 65–100%).

**Table 3.** Regions activated significantly more during theory-of-mind (ToM) reasoning as compared with physical reasoning (all *P* < 0.05, corr.)

	BA	*x*	*y*	*z*	*t*	No. of voxels
L superior temporal gyrus	38	−42	16	−20	9.13	295
R superior temporal gyrus	13	48	−41	20	9.00	556
L superior temporal gyrus	39	−45	−52	23	8.44	211
L precuneus	31	−3	−52	31	8.12	387
L cerebellum		−9	−34	−8	5.58	12
R fusiform gyrus	37	42	−40	−15	5.38	10
R medial frontal gyrus	9	6	50	15	5.25	8
L parahippocampal gyrus		−30	−8	−16	5.20	21
L thalamus		−9	−28	0	4.49	7
L parahippocampal gyrus		−33	−11	−16	4.41	6

### Visual perspective switching

Compared with baseline, both the Switch and Stick conditions of the perspective-switching task showed significant activation: the Switch condition was associated with activation of left posterior insula (−45, −7, 1; *t* = 4.16) and left STG (−31, 1, −14; *t* = 4.78), while the Stick condition indicated activation of right posterior insula (42, −4, 2, *t* = 5.14), left MFG (−21, −7, 46; *t* = 4.77), left IFG (−45, 25, −5, *t* = 4.25) and right transverse temporal gyrus (33, −27, 13, *t* = 4.08, all *P* < 0.05, corr.). However, no significant differences were evident in the direct contrast between the two conditions. Self-ratings for vividness of mental images were again high (*m* = 76.68, s.d. = 18.00), as were ratings of ease in making shifts in perspective (*m* = 75.53, s.d. = 21.85).

### Conjunctions of inner speech, theory-of-mind and perspective-switching

**Fig. 1.**
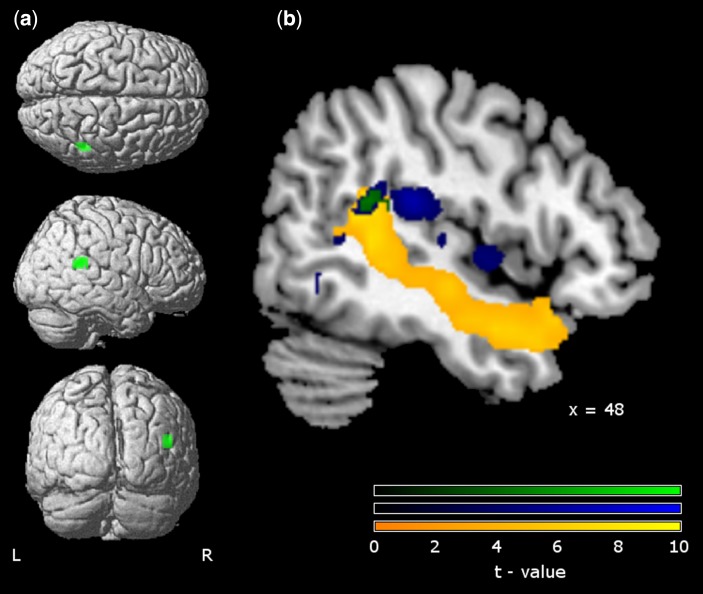
Conjunction of dialogic inner speech and theory-of-mind. A cluster in right STG (Fig.1a) was evident for both dialogic inner speech > monologic inner speech and ToM > physical reasoning, rendered here on the standard MNI brain supplied by SPM. Dialogic inner speech (Fig 1b; blue) was evident in right STG, cingulate and frontal gyrus, while ToM (yellow) was associated with extensive right STG activation running posterior to anterior. Their conjunction (green) was at the posterior end of right STG, in the TPJ area. ToM, Theory-of-mind; STG, superior temporal gyrus, all *P* < 0.05, corr., clusters > 6 voxels.

The contrasts between (i) dialogic and monologic inner speech and (ii) ToM and physical reasoning were incorporated into a conjunction analysis. As Figure 1 shows, only one cluster showed significant activation differences for both contrasts, centring on right posterior STG (48, −41, 20, *t* = 4.59, cluster size = 15, *P* < 0.05, corr.). Using a voxel-wise error of *P* < 0.01 for exploratory purposes, overlaps between the two tasks were also evident in right anterior STG, precuneus, right MTG, left paracentral lobule and right fusiform gyrus (all *P* < 0.05, corr.). When a conjunction analysis was run comparing the Dialogic > Monologic contrast and the Switch > Stick contrast, no significant clusters were observed (all *P* > 0.05, corr.). Overlaps at the lower significance threshold (voxel-wise *P* < 0.01) were evident in a ventral cluster encompassing the right posterior cingulate (3, −65, 10), the left cuneus (−12, −68, 7) and smaller clusters in right IFG (33, 31, 5) and left precuneus (−9, −60, 40).

### Individual differences in inner speech

We examined correlations between (i) Dialogic > Monologic inner speech activations and self-report scores for vividness during the task and (ii) Dialogic > Monologic inner speech activations and self-report scores on the VISQ. These analyses revealed very similar activation areas to the group analysis. Self-report scores for vividness of the inner speech scenarios were significantly associated with clusters in right posterior MTG (36, −58, 15; *t* = 5.47, cluster size = 37) and right cingulate gyrus (6, −23, 35; *t* = 4.93, cluster size = 9; *P* < 0.05, corr.). Scores on the Dialogic Inner Speech subscale of the VISQ were associated with a cluster in the same area of right MTG (39, −58, 15; *t* = 6.57, cluster size = 10), along with two areas of the right precuneus ((i) 15, −67, 26; *t* = 4.89, cluster size = 11; (ii) 15, −49, 31; *t* = 4.66, cluster size = 13; all *P* < 0.05, corr.). No significant associations were observed for self-reported use of visual imagery nor for the other components of the VISQ (evaluative, other people and condensed inner speech).

### Generating dialogic and monologic scenarios: the roles of inner speech and imagery processes

Phenomenological ratings from the mock scanner version of the task were used to examine use of inner speech and visual imagery across dialogic and monologic scenarios. As Table 4 indicates, dialogic and monologic scenarios were equivalent in all respects bar vividness of other voices (*t* = 7.47, *df* = 19, *P* < 0.001) and mean number of switches per scenario (*t* = 5.35, *df* = 19, *P* < 0.001), both of which were more common for dialogic inner speech (all *P* values are Bonferroni corrected). For both dialogic and monologic scenarios, inner speech was present to a significantly greater degree than visual imagery (dialogic: *t* = 3.21, *df* = 19, *P* = 0.036; monologic: *t* = 3.79, *df* = 19, *P* =0.010). As may be expected, vividness for one’s own voice was also stronger on average than vividness of other voices (dialogic: *t* = 5.95, *df* = 19, *P* < 0.001; monologic: *t* = 11.00, *df* = 19, *P* < 0.001).

**Table 4.** Self-reported vividness ratings for dialogic and monologic scenarios in mock scanner conditions

	Dialogic		Monologic		
	Mean	s.d.	Mean	s.d.	Sig.
Vividness (overall)	62.45%	11.73%	63.00%	13.00%	
In inner speech?	70.57%	19.23%	72.55%	22.37%	
In visual imagery?	46.08%	20.76%	38.05%	22.84%	
Vividness of own voice	69.90%	14.49%	73.40%	14.67%	
Vividness of other voices	43.91%	20.76%	18.80%	17.60%	***
Number of switches	1.65	1.07	0.40	0.59	***

***P < 0.001 (Bonferroni-corrected *P* values used).

Finally, Table 5 shows mean ratings for dialogic and monologic scenarios combined, compared across the normal and articulatory suppression versions of the task. Articulatory suppression had the effect of lowering vividness ratings for inner speech, one’s own voice and other voices but had no effect on levels of visual imagery (*P* = 0.999) and number of switches (*P* = 0.148).

**Table 5.** Self-reported vividness ratings for inner speech scenarios in mock scanner under normal conditions and during articulatory suppression

	Normal conditions		Articulatory suppression		
	Mean	s.d.	Mean	s.d.	Sig.
Vividness (overall)	62.73%	11.93%	36.01%	16.54%	***
In inner speech?	71.56%	20.18%	32.12%	23.38%	***
In visual imagery?	42.07%	20.90%	44.60%	22.18%	
Vividness of own voice	71.65%	14.31%	32.78%	20.77%	***
Vividness of other voices	31.36%	20.90%	15.88%	14.12%	**
Number of switches	1.03	0.69	0.55	0.49	

**P < 0.01, ***P < 0.001 (Bonferroni-corrected *P* values used).

## Discussion

This study attempted to examine neural differences between two varieties of internal self-talk: dialogic and monologic inner speech. In line with the hypothesis that generating dialogic scenarios would be associated with recruitment of a network extending beyond the left frontotemporal language regions, dialogue was associated with significantly greater activation, compared with monologue, in the precuneus, posterior cingulate and the right STG (BA13 and BA41), alongside activation in left insula, IFG, STG and cerebellum. Conjunction analysis identified an overlap with ToM reasoning specifically in right posterior STG, although shared substrates with visual perspective-switching could not be fully assessed due to null results in the contrast between switching and single-perspective imagery on that particular task.

The involvement of a left-hemisphere network including IFG, STG and the cerebellum during generation of dialogic scenarios is consistent with prior inner speech studies (Shergill *et al*., 2001; Simons *et al*., 2010; Geva *et al*., 2011) and implies a greater demand on these areas when a dialogue must be produced (in contrast to a monologue). Although the IFG and insula are often implicated in inner-speech tasks (although see Jones, 2009), activations of posterior STG and lateral regions of temporal cortex are observed depending on specific task demands, such as self-monitoring of inner speech rate (Shergill *et al*., 2002) and phonology (Aleman *et al*., 2005). The cerebellum, in contrast, has been proposed to support maintenance of verbal working memory (i.e. articulatory rehearsal) via its connections with motor cortex (Marvel and Desmond, 2010).

Although a number of right-hemisphere regions were active during the dialogic condition, there was less evidence to suggest that this involved the specific recruitment of either language region homologues or voice-selective areas. For example, although activation in the right STG was more anterior than in the left STG, and was close to regions that have been previously related to listening to familiar voices (Shah *et al*., 2001), it actually overlapped more with areas previously associated with spatial rather than auditory processing (Ellison *et al*., 2004). This suggests that the right-hemisphere differences between the dialogic and monologic conditions were not simply picking out additional voice representation demands (cf. Shergill *et al*., 2001) but relate instead to other cognitive factors.

The results of conjunction analysis indicated the involvement of social-cognitive processes in dialogic scenarios. Activity in posterior right STG was evident during both dialogic scenarios and ToM reasoning, in a region previously linked to both ToM (Fletcher *et al*., 1995) and imagery for personal perspectives (Ruby and Decety, 2001). It is also close to sections of right TPJ that have been implicated in representation of other people’s beliefs and states of knowledge (Saxe and Powell, 2006; Sebastian *et al*., 2012). Along with ToM, right TPJ has been proposed to play a role in managing divided attention and non–ToM-based perspective switching (Mitchell, 2008; Aichhorn *et al*., 2009), although there is debate as to whether these functions are subserved by the same or separable components of the TPJ (Scholz *et al*., 2009). Recent research on structural connectivity suggests that TPJ splits into three separate subregions: a dorsal component connecting to lateral anterior PFC, an anterior region connecting to the ventral attentional network and a posterior region connecting to social cognitive areas such as the precuneus and posterior cingulate (Mars *et al*., 2012). The cluster identified in this study would appear to be located between the latter two putative sub-regions of the TPJ, implicating both social-cognitive and attentional processes.

Apart from right STG, there was evidence (at less conservative significance levels) of functional overlaps between dialogic inner speech and ToM in an area of right MTG that has been previously linked to retrieval of face-word associations (Henke *et al*., 2003) and reflection on third-person traits (Kjaer *et al*., 2002). There was also overlap in posterior midline structures, although generally the two processes appeared to involve separate parts of the precuneus and posterior cingulate cortex, with the ToM cluster much closer to the midline. Dialogic inner speech also prompted activation in anterior medial frontal gyrus but ToM reasoning did not (cf. Walter *et al*., 2004).

The involvement of anterior and posterior midline structures in the contrast between dialogic and monologic conditions may indicate that the default mode network (DMN) is involved in generating internal dialogue (Buckner *et al*., 2008). ToM, autobiographical memory and resting-state cognition have been proposed to draw on a shared ‘core’ network including mPFC, precuneus, posterior cingulate and TPJ (Spreng *et al*., 2009). If the dialogic quality of inner speech imbues it (compared with monologic inner speech) with qualities of open-endedness, flexibility and creativity (Fernyhough, 1996, 2009), then it would arguably draw on some of the same introspective processes that the DMN is thought to underpin.

The remaining clusters identified in the contrast between dialogic and monologic inner speech also point to a range of processes associated with DMN functioning. Left precuneus has been associated with the simulation of third-person perspectives (Ruby and Decety, 2001) and episodic memory retrieval (Zysset *et al*., 2002), while right posterior cingulate has been linked to retrieval of autobiographical memories (Fink *et al*., 1996; Ryan *et al*., 2001). One possibility is that dialogic scenarios simply place a greater demand on memory processes, requiring the representation of specific events or people that would otherwise not be needed for generating one’s own voice. This seems unlikely, however, given that the monologic and dialogic scenario pairs were chosen to have the same general content (a school visit, a job interview, etc.), which should have minimized any differences between the conditions in terms of autobiographical memory demands. Alternatively, it may be that the scene construction processes thought to underpin autobiographical memory retrieval (Hassabis and Maguire, 2009) are similar to those recruited in producing a realistic and immersive dialogue. A direct comparison of scene construction, autobiographical memory and inner speech would be required to parse out these possibilities.

The results from the individual differences analysis highlighted a slightly different range of activation foci to the group contrast for dialogic > monologic inner speech: specifically, vividness ratings correlated with activation in the right MTG and cingulate gyrus, while dialogic inner speech (assessed as a general trait) correlated with the same MTG area, plus two sections of right precuneus. This contrasts with the involvement of ‘classic’ inner speech areas (left IFG, STG and cerebellum) and the focus on right STG seen in the group analysis of dialogic *vs* monologic inner speech.

The lack of correlates in the individual differences analysis in left frontotemporal areas suggests that covert articulation, *per se*, may not be so important for generating particularly vivid or dialogic scenarios. Nevertheless, the other areas identified in this analysis implicate similar processes and networks to the group analysis. For instance, the right MTG and the two sections of the right precuneus that correlated with dialogic inner speech reports have previously been implicated in theory-of-mind (Atique *et al*., 2011; Brüne *et al*., 2011). Other regions identified in this analysis are associated with processes that are also likely to be involved in generating dialogic scenarios. For example, right MTG has been associated with accurate and confident recall (Chua *et al*., 2006, Giovanello *et al*., 2010), while the right precuneus has been associated with retrieval of verbal episodic memory (Fernandes *et al*., 2005), context-rich autobiographical memories (Gilboa *et al*., 2004) and first-person perspectives memories (sometimes called ‘field’ memories; Nigro and Neisser, 1983; Eich *et al*., 2009). The activation of cingulate gyrus for vividness ratings, though likely not specific to this process, has been linked previously to a right anterior insula network involved in affective engagement (Touroutoglou *et al*., 2012). When these results are taken together, it might suggest that the tendency to engage in dialogic inner speech in everyday life does not reflect a trait towards ‘more’ inner speech—understood simply as a greater frequency of covert articulation—but instead indicates a greater tendency to recall and re-engage in previous interactions with others, and perhaps even to use these episodic memories to plan future social interactions.

### Limitations

One-key limitation in interpreting the present results is the extent to which the inner-speech task actually elicited inner speech. Participants were prompted to generate dialogic and monologic scenarios in inner speech, but they may have varied in their ability to do so, or may have drawn on other forms of simulation (such as visual imagery). Similar imagery-generation paradigms have been criticized in related fields (e.g. auditory imagery; Zatorre and Halpern, 2005) and in general it is preferable to include an objective test of inner speech use, such as paradigms that require participants to make rhyming judgements (Geva *et al*., 2011) or to assess metric stress (Aleman *et al*., 2005).

To address this limitation, we gathered behavioural data from a mock scanner task in which a separate set of participants reported on their imagery processes for each scenario used during scanning. Scenario stimuli generally prompted high levels of inner speech compared with visual imagery across both dialogic and monologic scenarios, while both kinds of scenario proved difficult to generate (in the sense of leading to post-scan reports of vivid auditory imagery) when inner speech was blocked via articulatory suppression (repeating days of the week). Additional corroboration of the paradigm was provided by the individual differences analysis of inner speech scores, which implicated broadly similar brain regions (right posterior temporal and midline structures) and similar processes (Theory-of-Mind, autobiographical recall) to the main dialogic–monologic contrast.

Taken together, these data at least partly address the concern that participants did not engage in inner speech in producing dialogic and monologic scenarios. Nevertheless, the results presented here need to be replicated alongside a battery of other inner speech measures that do not rely on participants’ self-reports (Aleman *et al*., 2005), to fully assess the extent to which our new paradigm elicits dialogic and monologic inner speech. The individual difference correlates in particular require replication in a much larger sample than tested here.

A second limitation is that the perspective-switching task did not produce consistent activation maps that could be used in the conjunction analysis, thus limiting the assessment of whether the dialogicality of inner speech depends purely on demands associated with generating and coordinating mental imagery. A novel imagery task was deployed here to match the structure and timing of the inner-speech task but it is possible that a different task with similar demand characteristics would have provided a better control. For instance, mental rotation tasks involve demands to generate and flexibly manipulate mental images, and are consistently associated with activation in a network of frontoparietal regions (McNorgan, 2012).

### Implications for psychopathology

Notwithstanding these caveats, the results presented here could have important implications for understanding inner speech in both typical and atypical populations. Although the involvement of ToM-related networks in internal dialogue is perhaps unsurprising, our conjunction analysis findings align with the view that articulating different perspectives may be an important feature of more complex forms of inner speech (Fernyhough, 1996). Abnormalities in the interplay between inner speech and ToM networks may thus explain some important findings in atypical groups.

As a first example, dominant models of AVH explain the phenomenon in terms of misattributed inner speech but struggle to explain why these hallucinations are distinctly experienced in another person’s voice (Jones and Fernyhough, 2007). Previous work has already suggested that dialogic conceptions of inner speech may account for the presence of the voices of others in one’s head (Fernyhough, 2004). Our present study extends this by showing commonalities between many of the neural regions activated during AVH (such as left STG, left insula, left IFG) and those strongly activated during dialogic inner speech (Jardri *et al*., 2011; Kühn and Gallinat, 2012). Future studies should test the proposal that findings from AVH research can be accounted for by dialogic inner speech occurring in conjunction with altered activity in other neural areas, such as the SMA (McGuire *et al*., 1995; Raij and Riekki, 2012), causing it to be experienced as non–self-produced. Our study also implies that neuroscientific studies of AVH need to consider social-cognitive networks alongside speech processing to fully understand how such hallucinations occur (see also Bell, 2013).

As a second example, atypical ToM has for a long time been considered a core feature of autism spectrum disorder (Baron-Cohen *et al*., 1985), but differences in inner speech in autism have only been studied relatively recently (Whitehouse *et al*., 2006; Wallace *et al*., 2009; Holland and Low, 2010). Early experience in autism is characterized by delays in language development and significant difficulties with social and communicative interaction (WHO, 1993). If inner speech is shaped by communicative experience—as a Vygotskian approach would suggest—then qualitative differences in the inner speech of people on the autistic spectrum may also be expected (Fernyhough, 1996; Williams *et al*., 2012). The data presented here are consistent with the idea that there are important interconnections between atypical ToM skills and atypical inner speech, which may mutually inform one another over the course of development. The direction of this relationship remains to be explored: on the one hand, problems with ToM could cause a qualitatively different experience of inner speech in autism; on the other hand, a lack of conversational or communicative inner speech might impact ToM development through limiting opportunities for dialogic interaction with others (Fernyhough, 2008).

In conclusion, we have presented the first neuroimaging study of some important varieties of inner speech, focusing on the contrast between dialogic and monologic forms of self-talk. Our findings provide initial support for the idea that forms of inner speech exist which can be both phenomenologically and neurologically distinguished from the silent commentary of a single inner voice. The data presented here suggest that generating silent dialogues draws on a wider network than classical regions associated with language production and comprehension, including recruitment of a core part of the ToM network. Further work is needed to disambiguate (i) the exact processes shared between dialogic inner speech and ToM, (ii) the involvement of the DMN in this conjunction and (iii) relative contributions of inner speech and forms of mental imagery to creating vivid inner dialogues.

## Supplementary Material

Supplementary Data
