# Supplementary material for: The brain’s conversation with itself: neural substrates of dialogic inner speech
Source: Soc Cogn Affect Neurosci. 2015 Jul 20;11(1):110–20. doi: 10.1093/scan/nsv094 (PMC4692319; doi:10.1093/scan/nsv094)
Supplement: Supplementary Data [file supp_11_1_110__index.html]

The Brain’s Conversation with Itself: Neural Substrates of Dialogic Inner Speech — The brain’s conversation with itself: neural substrates of dialogic inner speech — The brain’s conversation with itself: neural substrates of dialogic inner speech — Supplementary Data 

# The brain’s conversation with itself: neural substrates of dialogic inner speech

## Supplementary Data

files

- Supplementary Data - docx file
