# Supplementary material for: The brain’s conversation with itself: neural substrates of dialogic inner speech
Source: Soc Cogn Affect Neurosci. 2015 Jul 20;11(1):110–20. doi: 10.1093/scan/nsv094 (PMC4692319; doi:10.1093/scan/nsv094)
Supplement: Supplementary Data [file supp_nsv094_scan-14-450-File008.docx]

**SUPPLEMENTARY MATERIAL**

**i) Example response sheet for mock scanner inner speech task.**

1. The first scenario asked you to imagine you were going back to your old school or college to meet one of your favourite teachers and have a conversation. Please rate the following criteria:

a) On a scale of 0-100, how vivid was the scenario you imagined?

(0 = Not at all, 100 = As vivid as a real conversation).

b) How much of the scenario involved inner speech, i.e. imagining words and sentences?

(0 = Not at all, 100 = All of it).

c) How much of the scenario involved visual imagery, i.e. imagining people’s faces, surroundings?

(0 = Not at all, 100 = All of it).

d) How vivid was your own voice during the scenario?

(0 = Not at all, 100 = As vivid as if I was speaking out loud now)

e) How vivid were the voices of other people during the scenario?

(0 = Not at all, 100 = As vivid as if there were speaking out loud now)

f) Did you switch between different voices or perspectives during the scenario?

YES/NO

If so, how many times do you think you did this? 1-2 3-4 5-6 7-8 8+
